# Epithelial-to-mesenchymal transition in the development of endometriosis

**DOI:** 10.18632/oncotarget.16472

**Published:** 2017-03-22

**Authors:** Yan-Meng Yang, Wan-Xi Yang

**Affiliations:** ^1^ The Sperm Laboratory, College of Life Sciences, Zhejiang University, Hangzhou, China

**Keywords:** epithelial-to-mesenchymal transition, endometriosis, infertility

## Abstract

Endometriosis, an estrogen-dependent chronic gynecological disease, is common in reproductive-age women and profoundly affects their life quality. Although various pathogenic theories have been proposed, the origin of endometriosis remains unclear. Epithelial to mesenchymal transition (EMT) is a process that epithelial cells lose polarized organization of the cytoskeleton and cell-to-cell contacts, acquiring the high motility of mesenchymal cells. These changes are thought to be prerequisites for the original establishment of endometriotic lesions. However, no study exactly indicates which type of EMT occurs in endometriosis. In this review, we conclude that two different types of EMT may participate in this disease. Besides, two stimulating signals, hypoxia and estrogen, can through different pathways to activate the EMT process in endometriosis. Those pathways involve many cellular factors such as TGF-beta and Wnt, ultimately leading to cell proliferation and migration. As infertility is becoming a serious and intractable issue for women, EMT, during the implantation process, is gaining attention. In this review, we will describe the known functions of EMT in endometriosis, and suggest further studies that may aid in the development of medical therapy.

## INTRODUCTION

Endometriosis, an estrogen-dependent chronic gynecological disease, is defined as the presence of functional endometrial stroma and glands outside the uterine cavity. The disease affects 10%-15% of woman in reproduction age [1], often associated with chronic pelvic pain and infertility [2]. There are three types of endometriotic lesions: peritoneal, ovarian endometriosis and rectovaginal, which are classified by the different location of lesions, may have different pathogeneses [3]. However, study has found that all endometriotic lesions would share the same commonality as being the repeated tissue injury and repair (ReTIAR), resulting in fibrosis [4]. Endometriosis is considered as a multifactor disease affected by hormonal, immunological, and environmental factors, while lacking a clear Mendelian pattern of inheritance. Recent molecular cytogenetic studies provide novel evidence that acquired chromosome-specific alterations may induce endometriosis [5]. But strong gene-environmental interactions might definitively influence the approaches to identify genetic variants involved [6]. So even endometriosis has long been studied, the exact cause of this disease is still unclear.

There are various theories that have been proposed to explain the development of endometriosis. The most widely accepted theory is the retrograde menstruation or transplantation theory [7]. Recently, a new branch of this theory implicates that mesothelial epithelial to mesenchymal transition (EMT) as the pathogenic mechanism [8]. A patch of mesothelial cells that has undergone EMT no longer provides the sheet-like protective barrier between the basal layer and the luminal space. Absent of the mesothelial barrier, endometrial cells could easily adhere to the underlying peritoneal stroma and establish endometriotic lesions. In both peritoneal and ovarian endometriotic lesions, epithelial markers were found downregulated in the epithelial cells, while the mesenchymal markers upregulated [9, 10]. Besides the change of cell makers, there are several other evidences indicating that EMT is essential for the development of endometriosis. For example, one of the challenge of Sampson's retrograde hypothesis is that cells would die when they detach from extracellular matrix (ECM) or adhere to inappropriate locations, which named as anoikis. However, EMT has a main feature associated with anoikis resistance, contributing to the spread of ectopic lesions [11]. Secondly, during the development of the urogenital system, endometrium is derived from intermediate mesoderm *via* mesenchymal to epithelial transition (MET). As some imprint of their mesenchymal origin retained, endometrial epithelial cells may have a particularly tend to return to the original state, *via* EMT [12].

However, since EMT process is mainly studied in cancer field and the origin of endometriosis is controversial, there are few investigations of EMT in endometriosis. Nowadays, the related work is still stagnant on tissue level, no accurate EMT signal pathways have been studied in endometriotic cells. So far majority of studies didn't explain which type of EMT exactly occurs in endometriosis. Based on existed literature research, we conclude that two different types of EMT may be involved in. This hypothesis urgently need further experimental evidences, and it may provide a new therapy target for this disease. Besides, two stimulating signals, hypoxia and estrogen, can through different pathways to activate the EMT process in endometriosis. Those pathways involve many cellular factors such as TGF-β and Wnt, ultimately leading to cell proliferation and migration. At last, we are trying to explain the infertility, a severe problem caused by endometriosis, through an EMT related aspect and provide a potential therapy implication. We hope this relative comprehensive review of EMT in endometriosis could appeal to more investigations, ultimately lead to radical treatments for this disease.

## DIFFERENT TYPES OF EMT ARE INVOLVED IN ENDOMETRIOSIS

At a 2007 meeting on EMT in Poland and a subsequent meeting in March 2008 at Cold Spring Harbor Laboratories, EMT is classified into three different biological subtypes based on the biological context in which they occur. Type 1 EMT occurs during embryo development both in vertebrates and invertebrates, involved in the generation of tissues and organs [13]. It is an entirely normal physiological process and not associated with other abnormal function such as inflammation, fibrosis, or invasion; Type 2 EMT, often occurs in response to wound or inflammatory injury [14]. When the injury is acute, Type 2 EMT will be limited. However, under chronic injury condition, the damage and inflammatory response are persistent, Type 2 EMT would contribute to tissue fibrosis and other organ destructions; Type 3 EMT is recognized as the major cause of the tumor metastatic. With the induction of angiogenesis, Type 3 EMT can strongly promote the cancer cells to undergo a series of steps characteristic of metastatic cascade [15, 16]. Another way to classify different types of EMT is meta-analysis of multiple microarray datasets. The three previous lists of EMT-related genes are referred as PO-List [17], EM-list [18] and SC-list [19]. However, low overlap of the generic signatures generated from different investigations suggests that generic network employed by EMT could be more complex than expected [20]. In this review, we will follow the classic three types of EMT theory, which is more widely acknowledged.

**Figure 1:**
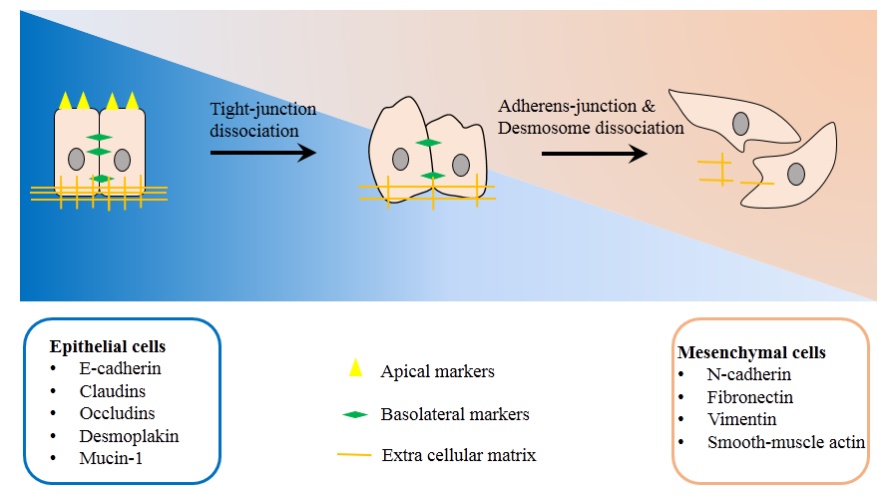
Basic mechanism of Epithelial-to-mesenchymal transition Epithelial cells gradually loss cell-to-cell junctions and degrade extra cellular matrix, finally transformed into mesenchymal cells. Phenotype and functional markers of EMT include: Spindle shape to fibroblast like phenotype; losing of epithelial markers such as E-cadherin, Desmoplakin, Mucin-1, occludin, and claudin; gain of mesenchymal markers such as N-cadherin, smooth-muscle actin, vimentin, and fibronectin et al; increased invasion capacity; increased resistance to anoikis/ apoptosis.

EMT is a biology process that cells lose the epithelial features and instead gain properties of mesenchymal cells. This process requires a series of complex changes in cell architecture and behavior, which often driven by the various cellular signals (Figure 1). The molecular change correlated with this transition include the loss of other epithelial markers such as E-cadherin, Desmoplakin, Mucin-1, occludin, and claudin, and the gain of mesenchymal markers such as N-cadherin, smooth-muscle actin, vimentin, and fibronectin et al. All of these molecular changes are associated with the alteration of cell functions such as enhanced migration, invasiveness and resistance to apoptosis [21, 22].

Transcription factors such as the Snail family of zinc-finger transcription factors (Snail, Slug, and Smuc), the δEF1 family of two-handed zinc-finger factors (δEF1/ZEB1 and SIP1/ZEB2), and the basic helix-loop-helix factors Twist and E12/E47 are known as the key EMT regulators [23]. Except for Twist, these transcription factors could repress expression of E-cadherin by directly binding to the E-box sites in its promoter [24]. MicroRNAs are also tightly connected with the regulatory network of those transcriptions. For example, members of the miR-200 family maintain an epithelial status and prevent EMT through inhibition of ZEB1 and ZEB2 [25]. In turn, miR-200 members are transcriptionally repressed by ZEB factors—as well as Snail1—thus forming a double-negative loop that maintain cells in either an epithelial or mesenchymal state [26]. P53 could down-regulate Snail1 and Snail2 *via* induction of miR-34a/b/c. [27] Conversely, Snail1 and Snail2 (and ZEB1) transcriptionally repress miR-34a/b/c [28]. These double-negative loops regulate the transition direction of cells, and well explained the complex of EMT process. These cellular changes are almost common features in all three EMT types, which make the specified study of each type more difficult.

Though the detailed differences governing the three EMT types are not yet clear, their functional distinctions are apparent. Considering endometriosis is a chronic gynecological disease and has a relative risk deteriorating to tumor, we speculate that Type 2 and Type 3 EMT might be involved in this process. As mentioned above, endometriosis is a ReTIAR process and is histologically characterized by dense fibrous tissue surrounding the lesions [29]. This is more distinct in deep infiltrating endometriosis, and about 10%-15% of endometriosis cases formed dense scarring [30]. Besides, endometriosis has long been recognized as an inflammatory disease, for dysregulation of immune response has been noted in patients [31, 32]. Based on those evidence, we can infer that Type 2 EMT might be involved in the fibrosis of endometriosis, by participating the chronic inflammatory reaction. On the other hand, one of the most specific feature that depart the endometriosis from other benign disease, is the metastasis ability as malignancy induced by Type 3 EMT. This enhanced metastasis ability is often related with angiogenesis, which promotes endothelial functions, vascular permeability and development of experimental endometriosis [33].

As it's not enough to focus on part of characteristics of endometriosis to design effective therapeutics, further studies need to pay attention to the different types of EMT in different phase of the endometriosis.

## TWO STIMULATING SIGNALS MAY INDUCE EMT IN ENDOMETRIOSIS

### Hypoxia signal

The hypoxic conditions is marked by a transcriptional response of the exposed cells that result in upregulated expression of hypoxia-inducible factors (HIFs). HIF-1 is the most characterized regulator of the cellular response to hypoxia, composed of HIF-1α and HIF-1β subunits. In contract to the constitutively expressed HIF-1β subunit, the expression and activity of the HIF-1α subunit are precisely regulated by the cellular O2 concentration [34]. In normal, eutopic and ectopic endometrial tissues, overexpression of HIF-1α and morphology changes associated with EMT were observed [35]. Hypoxic and ischemic conditions can induce the EMT of endometrial cells, not only in the healthy endometria, but also the eutopic endometria of patients with endometriosis [36]. According to the retrograde menstruation theory, we can hypothesis that endometrial epithelial cells might be adapted to specific microenvironments like hypoxia during implantation, and the enhanced invasion capacity by EMT can contribute to the development of ectopic lesions. Obviously, this process also requires angiogenesis, which is the most essential process induced by hypoxia in the endometriotic lesions. Vascular endothelial growth factor (VEGF) is strongly expressed in both in eutopic and ectopic lesions [37], and an increased level of VEGF was found in the peritoneal fluid of women with endometriosis [38]. The VEGF/NRP-1 axis may also be associated with enhanced EMT process and the NF-κB and β-catenin signaling in tumor metastasis [39]. Subsequent to HIF-1α and VEGF-dependent pathway, Reactive oxygen species (ROS) are found to stimulate EMT and increased invasion capability in several human cancer cells [40, 41]. Higher endogenous oxidative stress was found in endometriotic cells and could lead to an increase of ROS production and a drop in catalase levels [42]. In addition, lysyl oxidases isoforms (LOX and LOXL2) are also suggested as EMT mediators of hypoxia, which probably promote epithelial cell plasticity by functional interacting with certain transcriptional repressors. A study demonstrated that higher LOXs was expressed in endometriotic lesions compared with normal endometrium [43]. It should be noted that several other factors related to EMT process were up-regulated under hypoxia, including HGF, NF-κB, Tswist1 and TGF-β [44, 45]. Moreover, the Notch signaling pathway was also involved in hypoxia-induced EMT by increasing motility and invasiveness [46].

### Estrogen signal

As a hormone dependent disease, endometriosis is deeply regulated by estrogen-mediated cellular signaling. Compared with normal endometrium, endometriotic lesions exhibit upregulated estradiol biosynthesis and low level of estradiol inactivation [47]. Here are two different forms of the estrogen receptor (ER), ERα and ERβ, independently encoded by two different gene, ESR1 and ESR2. Each ER isoform has a unique expression pattern in the endometriotic tissues. Indeed, in the female reproductive system, ERα is expressed primarily in the uterus, whereas ERβ is expressed primarily in the ovary [48]. In the previous studies, people believed that whether of ovarian or peritoneal locations, had higher ERβ expression rather than ERα compared with normal human endometrial cells [49], which was similarly detected in many animal models of endometriosis [50, 51]. However, a recent study shows that ERα expression was increased in ovarian endometriotic lesions compared with normal endometrium [52]. Those complicated results suggest that more detailed works should be carried out to understand the exact mechanisms of ERs action in endometriosis progression. And here we will temporally focus on the roles of both ERα and ERβ in the EMT process.

Firstly, ERα could directly bind to hepatocyte growth factor (HGF) promoter, then induces EMT in the human endometrial epithelial cells [53]. Besides, ERα could also possibly up-regulate of both Snail and Slug through activation of their promoter activities [54]. In the case of ERβ, no direct research has shown that it can regulate EMT in the endometriosis. But in other disease especially the cancer, the expression of ERβ usually negatively related to EMT process. For example, in the triple-negative breast cancer, ERβ1 could bind to and oppose the transcriptional activity of mutant p53 at the promoters of genes that regulate metastasis [55]. In basal-like breast cancer cells, the inhibition of EMT correlates with an ERβ-mediated upregulation of miR-200a/b/429 and the subsequent repression of ZEB1 and SIP1, which results in increased expression of E-cadherin [56]. However, not in all the conditions ERβ could inhibit EMT process, for Lung Adenocarcinoma as a contract example [57].

**Figure 2:**
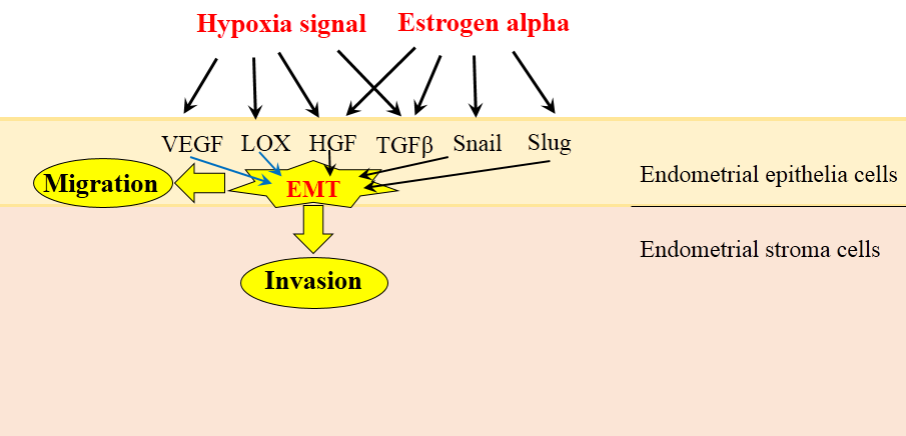
Hypoxia and Estrogen signals stimulate EMT in endometrial epithelial cells Hypoxia and Estrogen are two major stimulating signals found in endometriotic lesions, both could activate various cellular factors, such as HGF, TGF-β, VEGF, LOX, Snail and Slug, then induce the EMT process in endometrial epithelial cells (black arrows). The role of VEGF and LOX in EMT only been reported in cancer cells while no study in endometrial cells, so we use blue arrows to show them. Endometrial epithelial cells undergo EMT process will gain enhanced migration and invasion ability, which lead to the form of endometriotic lesions.

Hypoxia and Estrogen are two major stimulating signals found in endometriotic lesions, both contributing to EMT process through various pathways (Figure 2). As hypoxia condition has been found in majority cancer lesions, the studies of hypoxia in EMT are more abundant than estrogen signal. However, the high expressed estrogen is the unique feature of endometriosis, so the investigations of estrogen may provide a more effective approach to therapy. Meanwhile, these extracellular signals also could be used as effective methods to induce experimental endometriosis for study.

## BASIC CELLULAR PATHWAYS PROMOTE EMT PROCESS IN ENDOMETRIOSIS

### TGF-β/Smad

TGF-β gains more attention among EMT inducers, largely because of its potency in inducing EMT in cell culture and its roles in cancer and development associated EMT. The basic TGF-β signaling pathway starts through two types of serine-threonine kinase receptors, the TGFβRI and TGFβRII. The dimerized Receptors leads to the recruitment and phosphorylation of transcription factors Smad2 and Smad3, which could trimerize with Smad4 and then the complex translocates to the nucleus, ultimately regulates related gene transcription [58]. Smad could activate the transcription factors such as Snail1, Snail2/Slug, ZEB1, ZEB2, E12/E42 and Twist1, resulting in the loss of desmosome, cell-cell tight and adherens junctions, and the gain of mesenchymal markers. However, TGF-β also induces non-Smad signaling pathways, leading to activation of Rho GTPases, MAP kinase (MAPK) pathways and the PI3 kinase-Akt-mTOR pathway [59]. These non-Smad signaling pathways often cooperate with Smad-mediated gene expression during EMT, yet also directly regulate the stabilities and activities of Smads.

Up-regulated TGF-β in peritoneum and serum/peritoneal fluid in endometriotic tissues are frequently observed in patients with endometriosis [60, 61], which suggesting that they may be essential in establishment and/or maintenance of endometriosis [62]. Functionally, TGF-β1 could inhibit natural killer cell activity in the peritoneal fluid from women with endometriosis, and promote angiogenesis and cell proliferation in ovarian endometriotic cysts [63]. TGF-β1 could activate the expression of VEGF-A in the peritoneal mesothelial cell to support endometriotic lesions vascularization [64]. In animal mode, TGFβ1-null mice developed fewer and smaller peritoneal endometriosis-like lesions compared with their wild-type counterparts [65]. However, detailed mechanisms of how increased TGF-β concentrations contribute to endometriosis remains poorly understood.

Recently, study shows that pluripotent transcription factor OCT4, initiated by TGF-β signals, could in turn regulate the TGF-βI/TGF-βRI-induced the endometriotic lesions growth by stimulating endometrial cell migration. In human endometriotic stromal cells, TGF-βI dose-dependently increased the gene and protein levels of OCT4, SNAIL and N-Cadherin and silencing of endogenous OCT4 significantly suppressed the TGF-βI-induced expressions of N-Cadherin and SNAIL [66]. This finding demonstrate that TGF-β, cooperating with OCT4, may contribute to EMT process in endometriosis.

Additionally, TGF-β has long been regarded as a major regulator of fibrosis. For example, EMT and fibroblast-to-myofibroblast transition (FMT) induced by TGF-β1/Smad3 could result in increased collagen production, cellular contractility and smooth muscle metaplasia (SMM). The SMM has been frequently found in peritoneal, ovarian, extragenital, and pleuropulmonary endometriosis [67]. Another process abundant in peritoneal fibrotic tissue is mesothelial-to-mesenchymal transition (MMT), that mesothelial cells (MCs) in the peritoneal cavity could transform into myofibroblasts under pathological conditions. In mouse model, blockage of TGF-β resulted in molecular reprogramming of markers related to the mesenchymal conversion of MCs and in a significant decrease in the severity of the peritoneal adhesions [68].

### Wnt/β-catenin

Wnt/β-catenin signaling pathway is widespread in development, tissue self-renewal and various diseases including endometriosis. Β-catenin is a central intracellular transducer, which could bind the transcription factor/lymphoid enhancing-binding factor (TCF/LEF) family (TCF1, TCF3, TCF-4 and LEF) to regulate expression of hundreds of genes, including those that promote cell proliferation and survival [69]. In normal condition, the level of β-catenin in cytoplasmic is tightly controlled by GSK-3β phosphorylation, which triggers its degradation process through the ubiquitin pathway. Wnt-1 and Wnt-3 could inhibit the phosphorylation of GSK-3β thus preventing β-catenin from entering the ubiquitination pathway, thereby forming β-catenin pools [70]. The free cytoplasmic β-catenin could relocate into the nucleus to activate transcription factors LEF-1/TCFs, then induce the cells undergo EMT [71].

Many studies have revealed the association between Wnt signaling pathway and endometriosis. Significantly higher expression of *WNT7A* has been found in human endometriotic lesions, which may influence cell survival, promoting an improved implantation of scattered endometrial cells and hence an increase in formation of endometriosis lesions [72]. In addition, elevated secretion of Wnt2 in ectopic stromal cells could activate the growth of epithelial cells *via* Wnt2/β-catenin signaling pathway in a paracrine manner [73]. Estrogen could also up-regulate β-catenin expression by directly binding to the estrogen response element site on the promoter of β-catenin in endometriotic lesions [74]. Just like TGF-β, another role of Wnt/β-catenin signaling pathway in endometriosis is inducing fibrosis. Aberrant activation of the Wnt3/β-catenin was involved in mediating fibrogenesis in this disease. After the blocking of TCF/β-catenin pathway, the expression of myofibroblasts markers, such as α-SMA, collagen I and fibronectin are decreased in endometriotic stroma cells [75].

**Figure 3:**
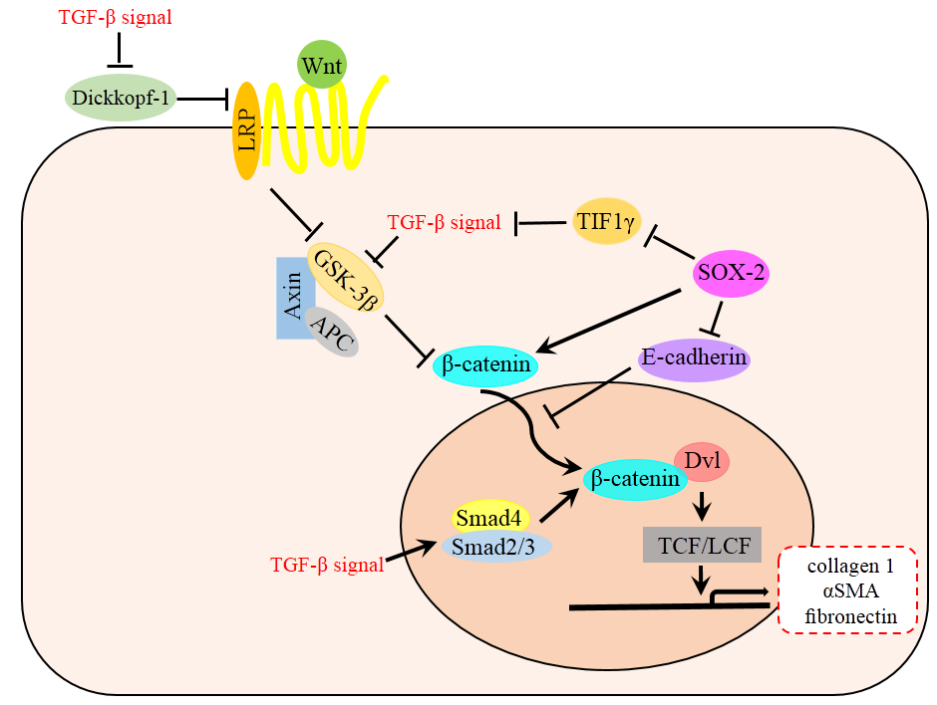
Relationship between Wnt and TGF-β signaling pathway in EMT TGF-β signal is involved in many steps of Wnt signaling pathway, which ultimately accumulate β-catenin and then promote the EMT and fibrosis. TGF-β mediates decreased expression of Wnt antagonist Dickkopf-1 *via* p38 dependent manner, meanwhile, TGF-β stimulates Wnt signaling by inhibiting GSK-3β or promoting Smad4 and Smad2/3 complex to increase β-catenin both in cytoplasm and nucleus. TGF-β signal is also activated by SOX2 through the repression of TIF1γ. Besides, SOX2 could promote the Wnt signaling pathway by transcriptionally increasing the expression of β-catenin and enhancing the nucleus relocation process.

TGF-β/Smad and Wnt/β-catenin pathways may exist cooperation in EMT and fibrosis. In renal tubular epithelial cells, hydrogen Sulfide could Inhibit TGF-β1-Induced EMT *via* Wnt/Catenin Pathway [82]. TGF-β could mediate decreased expression of Wnt antagonist Dickkopf-1 *via* p38 dependent manner, highlight a key role for the interaction of both pathways in the pathogenesis of fibrotic diseases [83]. TGF-β might also stimulate Wnt signaling by other mechanisms such as the inhibition of GSK-3β aside to increase β-catenin [84]. As a marker of endometriosis, SOX-2 participates in the network of TGF-β and β-catenin [85]. SOX2 could transcriptional repress the transcriptional intermediary factor 1 γ (TIF1γ), and therefore promote TGF-β-induced EMT and cell invasion [86]. SOX2 could also reduce the expression of E-cadherin at plasma membranes, thus reducing the binding capacity of the β-catenin complexes. Therefore, the Wnt signaling components in the nucleus could recruit more β-catenin to activate the downstream targets [87]. The complex processes of EMT requires us to take a network-wide view on signal transduction (Figure 3), and further studies about the relationship between Wnt/β-catenin and other EMT pathways are needed.

## EMT IN ENDOMETRIA MAY LEAD TO INFERTILITY

It has been estimated that at least one third of reproductive-age women would be affected with fertility problems [88]. Altered peritoneal function resulting in impaired folliculogenesis and oocyte quality, pelvic anatomy distortion, immunologic dysfunction, and impaired implantation are part of contributing factors to reduced fertility [89]. Besides, significantly reduced implantation rates are shown in women who received embryos from endometriotic ovaries [90].

Uterine receptivity, which allows the developing embryo to implant, is a complex process involving regulation by hormones, cytokines, adhesion molecules and other factors [91]. Normally at the time of implantation, estrogen receptors are downregulated; however, women with endometriosis have an upregulation of endometrial estrogen receptors [92]. Aromatase is also aberrantly expressed in the endometrium of women with endometriosis, increasing the amount of active estradiol [93]. Conversely, an increase of progesterone compared with estrogen must occur for successful endometrial receptivity to the implanting blastocyst. However, progesterone resistance has been reported in eutopic and ectopic endometrium [94]. EMT process is often associated with progesterone resistance, and progesterone could inhibit EMT by inactivating PI3K/Akt pathway in basal phenotype breast cancers [95]. This suggests that EMT process happened in endometriotic lesions may cause a low response of endometrial cells to progesterone, ultimately result in infertility. Embryo implantation is a continuous process initiated by interaction between the blastocyst and uterine luminal epithelium. Subsequently, the embryo forms stable adhesion with epithelial cells and then makes an invasion into endometrial stroma. Although the detailed mechanisms remain incomplete, it is well established that EMT plays a key role in this process [96]. However, based on insufficient evidence, we can't go to the conclusion that enhanced EMT in endometriosis could promote implantation. Because the expression of various molecules during embryo implantation was frequently changed in different phage, EMT is not always needed throughout the implantation. Meanwhile, the altered EMT in endometriosis is a pathological process, so it may have different ways from normal condition and lead to the failure of implantation.

## CONCLUSION

Studies on EMT in the development of endometriosis represented a limited amount of publications, and these findings are mainly concentrate in recent years. However, the phenomenon of EMT has long been found in endometriotic lesions. A number of studies focus on the invasion and fibrosis in endometriosis also showed the significance of EMT. Stimulating signals such as hypoxia and estrogen activate the origination and migration of endometriotic lesions. Basic pathways of EMT, such as TGF-β and Wnt, could provide potential therapeutic approach to inhibit endometriotic cell motility and invasiveness. The detailed mechanisms of these factors in the connection between EMT and endometriosis are still unclear, so further studies are needed. Infertility as the major threaten to the life of women, is tightly regulated by EMT process during implantation. At last, we hope this relative comprehensive review of EMT in the development of endometriosis could appeal to more investigations, ultimately lead to radically novel treatments for this disease.

## Abbreviations

EMT: epithelial-to-mesenchymal transition
TGF-β: TGF-beta
ReTIAR: repeat tissue injury and repair
ECM: extracellular matrix
MET: mesenchymal to epithelial transition
MSCs: Mesenchymal stem cells
VEGF: vascular endothelial growth factors
MAPK: Mitogen-activated protein kinase
FMT: fibroblast-to-myofibroblast transition
SMM: smooth muscle metaplasia
MMT: mesothelial-to-mesenchymal transition
MCs: mesothelial cells
HIFs: hypoxia-inducible factors
HGF: hepatocyte growth factor
TIF1γ: Transcriptional intermediary factor 1 γ.
